# Mariposa House service evaluation project & co-production: new women's NHS forensic community step-down hostel

**DOI:** 10.1192/bjo.2021.866

**Published:** 2021-06-18

**Authors:** Mandip Jheeta, Liam Dodge, Elizabeth Zachariah, Theresa Connolly

**Affiliations:** 1 Oxleas NHS Foundation Trust; 2 Oxleas NHS Foundation Trust; Expert-by-Experience, Oxleas NHS Foundation Trust; Expert-by-Experience AT, Oxleas NHS Foundation Trust; Expert-by-Experience AD, Oxleas NHS Foundation Trust

## Abstract

**Aims:**

To understand and learn from patients’ views and experiences. Ultimately, to improve quality, safety, and patients’ experiences and outcomes.

Service evaluation project of Mariposa House, London, the new women's forensic high support community step-down hostel after hospital admission. Run in partnership with Langley House (charitable) Trust. It is a co-produced, rare and innovative service- to our knowledge the only NHS women's service of its kind in England. In female and forensic community populations: transitions are the highest risk periods; the same treatment as men is unlikely to produce the same outcomes; and performance indicators and outcome measures are poorly understood.

**Method:**

Confidential patient questionnaire and self-reported Recovering Quality of Life (ReQoL) measure. Given to all patients in Mariposa House, before (in hospital) and 2-3 months after transfer to hostel. Themes included “my: care; voice (co-production); transition; & gender”. 12 questionnaires were received from 9 patients: 5 completed both pre- & post-; 3 (20%) were given but not received. Analysed by thematic content analysis. Additional focus group feedback session with patients and staff.

**Result:**

Overall, patients had very positive and similar views about both hostel and hospital(s), and similar views about both. Generally, patients feel treated with compassion, dignity and respect, and listened to and understood by staff members. They feel involved in and positive about their care.

There was a huge amount of involvement in co-producing the service and feeding back experiences, which has been very helpful. Co-production activities included: interviewing for staff and tenders; choosing hostel building; stakeholder meetings; and participating in meetings about training, policies and expectations. “I've been in hospital for so long moving was scary! But helping set up the project has given me confidence to move.”

There was strong agreement that transitions are difficult. Views on gender-specific needs being met were very positive, for both hostel and hospital. The main area for improvement was having better awareness of local neighbourhood and facilities- booklet now produced. Quality of life measures were at least maintained from hospital to hostel: 80% (n = 4) showed no reliable improvement/ deterioration, and 20% (n = 1) showed reliable improvement.

**Conclusion:**

There are very positive and similar views about the hostel and hospital(s). Co-production and service user involvement has been very helpful. The new hostel has maintained patient satisfaction and quality of life measures compared to established inpatient services. These are positive findings, and crucially: in a less- secure, contained, established, and cheaper new community setting, involving complex and challenging transitions.

